# Biomarker monitoring of controlled dietary acrylamide exposure indicates consistent human endogenous background

**DOI:** 10.1007/s00204-017-1990-1

**Published:** 2017-05-22

**Authors:** Katharina Goempel, Laura Tedsen, Meike Ruenz, Tamara Bakuradze, Dorothea Schipp, Jens Galan, Gerhard Eisenbrand, Elke Richling

**Affiliations:** 1 Division of Food Chemistry and Toxicology, Department of Chemistry, University of Kaiserslautern, Erwin-Schroedinger-Straße 52, 67663 Kaiserslautern, Germany; 2 ds-statistik.de, Pirnaer Straße 1, 01824 Rosenthal-Bielatal, Germany; 3 Hochgewanne 19, 67269 Grünstadt, Germany

**Keywords:** Endogenous acrylamide, Mercapturic acids, Human background, Duplicate diet study, Coffee, Hemoglobin adducts

## Abstract

The aim of the present study was to explore the relation of controlled dietary acrylamide (AA) intake with the excretion of AA-related urinary mercapturic acids (MA), *N*-acetyl-*S*-(carbamoylethyl)-l-cysteine (AAMA) and *N*-acetyl-*S*-(1-carbamoyl-2-hydroxyethyl)-l-cysteine (GAMA). Excretion kinetics of these short-term exposure biomarkers were monitored under strictly controlled conditions within a duplicate diet human intervention study. One study arm (group A, *n* = 6) ingested AA via coffee (0.15–0.17 µg/kg bw) on day 6 and in a meal containing an upper exposure level of AA (14.1–15.9 μg/kg bw) on day 10. The other arm (group B) was on AA minimized diet (washout, 0.05–0.06 µg/kg bw) throughout the whole 13-day study period. On day 6, these volunteers ingested ^13^C_3_D_3_-AA (1 μg/kg bw). In both arms, urinary MA excretion was continuously monitored and blood samples were taken to determine hemoglobin adducts. Ingestion of four cups of coffee resulted in a slightly enhanced short-term biomarker response within the background range of group B. At the end of the 13-day washout period, group B excreted an AAMA baseline level of 0.14 ± 0.10 µmol/d although AA intake was only about 0.06 µmol/d. This sustained over-proportional AAMA background suggested an endogenous AA baseline exposure level of 0.3–0.4 µg/kg bw/d. The excretion of ^13^C_3_D_3_-AA was practically complete within 72–96 h which rules out delayed release of AA (or any other MA precursor) from deep body compartments. The results provide compelling support for the hypothesis of a sustained endogenous AA formation in the human body.

## Introduction

Acrylamide (AA), a process-related food contaminant metabolically converted into a genotoxic carcinogen, has been classified as probably carcinogenic to humans (group 2A) (IARC 1994). It is formed during the Maillard reaction by heating of foods (>120 °C) from asparagine and reducing sugars. Human dietary AA exposure in Europe has been estimated to range from 0.4–1.9 μg/kg bw/d (mean) to 0.6–3.4 μg/kg bw/d (95th percentile) (EFSA 2015).

AA is metabolized by cytochrome P 450 2E1 to the genotoxic epoxide 2,3-epoxypropane amide (glycidamide, GA). GA induces DNA damage, predominantly by formation of *N* ^7^-GA-guanine adducts (Watzek et al. 2012a). Moreover, AA and GA react with plasma and other proteins, such as hemoglobin (Hb). Covalent binding to the *N*-terminal valine (Val) residue of Hb results in formation of *N*-(2-carbamoylethyl)valine (AAVal) and *N*-(2-carbamoyl-2-hydroxyethyl)valine (GAVal) Hb adducts that can be specifically cleaved off and derivatized to serve as biomarkers for long-term exposure monitoring (Bergmark et al. 1993; EFSA 2015; Fennell et al. 2005). AA as well as GA also reacts avidly with glutathione (GSH) to form GS-conjugates in the liver and potentially other metabolically competent organs. The GS-conjugates are further transformed to the corresponding *N*-acetylcysteine thioethers, the so-called mercapturic acids (MA) *N*-acetyl-*S*-(carbamoylethyl)-l-cysteine (AAMA), *N*-acetyl-*S*-(1-carbamoyl-2-hydroxyethyl)-l-cysteine (GAMA), and *N*-acetyl-*S*-(2-carbamoylethyl)-l-cysteine-sulfoxide (AAMA-sul) a human-specific metabolite (Fuhr et al. 2006; Wang et al. 2017; Watzek et al. 2012b). In a foregoing human intervention study (Ruenz et al. 2016), we investigated the relation between exactly controlled dietary AA intake, at about the mean European intake level and the output of the urinary exposure biomarkers, AAMA and GAMA. AA contents were determined in foods and liquids as consumed by preparing duplicates of meals, ready for ingestion. AA analysis in these diet duplicates ascertained an exact dosimetry of the AA intake by the volunteers. At the average intake level of European consumers (0.6–1.8 μg AA/kg bw/d), mean excretion of AAMA reached 58% of the ingested AA within 72 h, whereas about 7% were found to be excreted as GAMA. The design of this intervention study encompassed an initial 3-day period during which volunteers were on AA-minimized diet (washout period). During this initial washout period, dietary AA exposure (as measured in diet duplicates) did not exceed 0.04 µg/kg bw/d, less than 10% of present day lower bound mean consumer exposure. Yet, at the end of the washout period, an AAMA baseline level of 93 ± 31 nmol/d was recorded. After correction for the dietary AA uptake during the washout period, this suggested an estimated net baseline level corresponding to an AA exposure of 0.2–0.3 μg/kg bw/d. This background of unknown origin may be regarded as quite substantial when put into perspective with the average dietary AA exposure in Europe.

In a foregoing experimental study, Sprague–Dawley rats were kept for 2 weeks on an AA-minimized diet prior entering an extended dose–response study. During this extended AA washout period, they were found to excrete background levels of AAMA, indicative of an estimated AA exposure of about 0.6–0.7 μg/kg bw/d. This suggested that similar to humans, rats excrete urinary AAMA levels indicative of AA background exposure of unknown origin (Watzek et al. 2012a).

The aim of the present human intervention study was to substantiate the relation of exactly controlled dietary AA intake in humans with urinary MA biomarker excretion and to further substantiate the evidence for a potential background exposure to AA, as indicated from urinary exposure biomarker dosimetry. To that end, an intervention study under controlled environmental conditions was designed involving 12 human non-smoking male volunteers, randomly allocated into two arms. One arm (B) was on AA-minimized diet (washout) throughout the whole 13-day study period. On day 6 these volunteers received a single oral administration of stable isotope-labelled ^13^C_3_D_3_-AA (1 μg/kg bw). The other arm (A) ingested exactly controlled dietary intakes of AA (ascertained in diet duplicates) on days 6 and 10. In both arms, urinary MA excretion was continuously monitored. Blood samples were taken at start, day 7, and at the end of the intervention to monitor Hb-adducts. It was also intended to address the question whether a daily exposure level brought about by normal daily coffee consumption was detectable by short/long biomarker monitoring. In addition, peak exposure conditions should also be included to reflect exposure conditions that may result from high-temperature cooking in certain households.

## Materials and methods

### Chemicals

Acrylamide (AA), isotopically labelled acrylamide (^13^C_3_-2,3,3-D_3_-AA), and pentafluorophenyl isothiocyanate (PFPITC) were purchased from Sigma Aldrich (Steinheim, Germany). Deuterium-labelled AA (D_3_-AA) and the respective mercapturic acids, *N*-acetyl-*S*-(carbamoylethyl)-l-cysteine (AAMA), and *N*-acetyl-*S*-(1-carbamoyl-2-hydroxyethyl)-l-cysteine (GAMA) as well as the deuterium-labelled MAs, *N*-(acetyl-D_3_)-*S*-(carbamoylethyl)-l-cysteine (D_3_-AAMA), and *N*-(acetyl-D_3_)-*S*-(1-carbamoyl-2-hydroxyethyl)-l-cysteine (D_3_-GAMA) were obtained from Toronto Research Chemicals (Toronto, Canada). 1-(2-carbamoylethyl)-(S)-5-isopropyl-3-pentafluorophenyl-2-thiohydantoin (AAValPFPTH), 1-(R,S)-(2-carbamoyl-2-hydroxyethyl)-(S)-5-isopropyl-3-pentafluorophenyl-2-thiohydantoin (GAValPFPTH) as well as the deuterium-labelled analogue 1-(2-carbamoylethyl)-(S)-5-isopropyl-D_7_-3-pentafluorophenyl-2-thiohydantoin (D_7_-AAValPFPTH), and 1-(R,S)-(2-carbamoyl-2-hydroxyethyl)-(S)-5-isopropyl-D_7_-3-pentafluorophenyl-2-thiohydantoin (D_7_-GAValPFPTH) were available from former studies. All HPLC-solvents (acetonitrile, methanol, and acetic acid) were of HPLC–MS grade; other reagents were of analytical grade.

### Study design

**Fig. 1:**
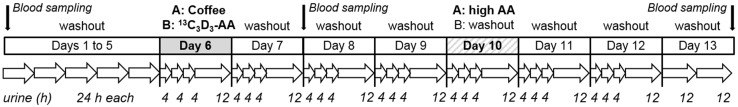
Study design of group A and B of the 13-day human intervention study; washout: maximum AA intake 0.05–0.06 µg/kg bw/d; coffee: AA intake 0.15–0.17 µg/kg bw/d; ^13^C_3_D_3_-AA: intake of 1 µg/kg bw of stable isotope labelled ^13^C_3_D_3_-AA; high AA: AA intake 14.1–15.9 µg/kg bw. Begin of each study day was 8 a.m. (0 h)

The intervention study was approved by the Ethics Commission of Rhineland-Palatinate [Mainz, no. 837.454.14 (9693)] and performed in accordance with the ethical standards laid down in the 1964 Declaration of Helsinki and its later amendments. Design, volunteer recruitment, and exclusion criteria were essentially the same as described before (Ruenz et al. 2016). In brief, 12 healthy male volunteers of Caucasian origin, non-smokers, and regular coffee consumers were recruited. Exclusion criteria were age <20 or >44 years, body mass index (BMI) <19 or >25 kg/m^2^, smoking or tobacco consumption, metabolic disorders, performing competitive sports, being under medication or taking dietary supplements, not being accustomed to regular coffee consumption, participation in other studies or regular blood donation. Prior to their inclusion in the study, all volunteers gave their informed consent. A medical check was performed to determine body weight, height, body mass index, blood pressure, and clinical parameters including general blood cell counts as well as creatinine, cystatin C, γ–glutamyltransferase (γ–GT), glutamate oxaloacetate transaminase (GOT), glutamate pyruvate transaminase (GPT), and C-reactive protein (CRP). All volunteers were of good health status with a BMI of 23.8 ± 1.1 kg/m^2^. The study location was a well-secluded hostel in the palatinate forest, with no open street traffic, no open fire, no tobacco smoking, nor any other known potential source for inadvertent AA exposure. Volunteers were under supervision 24 h/d. The design of the 13-day intervention study is shown in Fig. 1. Each study day started at 8.00 a.m. (0 h). Meals during the washout periods were identical for all volunteers (serving portions/time of ingestion) in both groups, and mineral water was allowed ad libitum during the entire study. After an initial washout period of 5 days on acrylamide-minimized diet (yoghurt, fruits, juice, vegetables, noodles, rice, and boiled meat), volunteers were divided into two groups of six each.

Group A: On day 6 the volunteers of group A consumed a washout meal and additionally 500 mL coffee throughout the day (0 h: 125 mL, 2.5 h: 125 mL, 6.5 h: 250 mL), whereas group B consumed hot water at the same time points. One cup of coffee (125 mL) was prepared from a coffee pad of 7.5 g (241 ± 11 μg AA/kg coffee powder). For group A the total AA intake was 12.1 μg (0.15–0.17 µg/kg bw), with 7.9 μg provided via coffee. Then three more washout days followed. On day 10, volunteers received a diet providing a high AA intake (high AA, 1140 µg), consisting of self-prepared French fries, commercially available potato crisps, crisp bread, cereals, Swedish oat cookies, and coffee as on day 6. Thereafter, volunteers of group A were on washout for another 3 days.

Group B: Volunteers were on the same washout regimen (AA minimized diet) throughout the whole study and received on day 6 a single oral administration of stable isotope-labelled ^13^C_3_D_3_-AA (1 μg/kg bw) in water. Furthermore, they ingested four cups of hot water (125 mL each) at the same time points (see above) as the volunteers of group A received the freshly brewed coffee.

For both groups total urine was collected during the whole intervention study, divided into different collecting periods as indicated (arrows) in Fig. 1. The total urine was weighed and stored at −20 °C until analysis. Blood samples were taken directly before study onset, at the end of day 7, and at day 13 (7:30 a.m.) from each volunteer immediately prior to breakfast. Weighed duplicates of all meals were homogenized and stored (−20 °C); coffee brew was frozen at −20 °C until analysis. Body weight of the volunteers was self-monitored daily (every morning before breakfast) and protocolled together with all physical activities.

### Experimental procedures

#### Food analysis (acrylamide, calorie content)

AA was determined in triplicate using a stable isotope dilution method (SIDA) as described by (Ruenz et al. 2016) with slight modifications. As internal standards, 250 ng D_3_-AA for foods with minor AA contents and 500 ng D_3_-AA for foods with higher AA contents were added. Subsequent sample work-up and analysis by high-performance liquid chromatography-electrospray-tandem mass spectrometry analysis (HPLC–ESI–MS/MS: Agilent 1290 HPLC system, Agilent Technologies, Waldbronn, Germany, coupled with a QTrap 5500 MS system, Sciex, Darmstadt, Germany) followed a previously published procedure except for HPLC–MS/MS analysis which was run with a reduced injection volume (1 µL). MS parameters are listed in Table 1. The calorie contents of all meal servings were calculated using the software Prodi 5.8 Expert (Nutri-Science GmbH, Hausach, Germany).

**Table 1:** Mass spectrometric (AB Sciex QTrap 5500) parameters to determine AA, AAMA, ^13^C_3_D_3_-AAMA, GAMA, AAValPFPTH, and GAValPFPTH in food and biological samples

	Q1	Q3	Time	DP	EP	CE	CXP	CUR	CAD	IS	TEM	GS1	GS2
AA	72.1	55.1	100^a^	36	10	16	15	40	Medium	4500	550	70	40
D_3_-AA	75.1	58.1	100^a^	36	10	15	7	40	Medium	4500	550	70	40
AAMA 1	233.0	104.0	3.9^b^	−30	−10	−20	−11	50	Medium	−4500	550	50	70
AAMA 2	233.0	162.0	3.9^b^	−30	−10	−14	−13	50	Medium	−4500	550	50	70
^13^C_3_D_3_-AAMA 1	239.1	110.0	3.9b	−40	−10	−20	−11	50	Medium	−4500	550	50	70
^13^C_3_D_3_-AAMA 2	239.1	162.1	3.9^b^	−40	−10	−15	−14	50	Medium	−4500	550	50	70
D_3_-AAMA	236.0	103.9	3.9^b^	−55	−10	−20	−5	50	Medium	−4500	550	50	70
GAMA 1	249.0	120.0	2.2^b^	−35	−10	−22	−11	50	Medium	−4500	550	50	70
GAMA 2	249.0	128.1	2.2^b^	−35	−10	−16	−9	50	Medium	−4500	550	50	70
D_3_-GAMA	252.0	119.7	2.2^b^	−30	−10	−20	−11	50	Medium	−4500	550	50	70
AAValPFPTH 1	394.1	275.1	160^a^	−110	−10	−30	−11	50	Medium	−4500	500	25	50
AAValPFPTH 2	394.1	302.9	160^a^	−110	−10	−22	−31	50	Medium	−4500	500	25	50
D_7_-AAValPFPTH	401.1	310.1	160^a^	−125	−10	−24	−17	50	Medium	−4500	500	25	50
GAValPFPTH 1	410.0	226.9	160^a^	−110	−10	−22	−11	50	Medium	−4500	500	25	50
GAValPFPTH 2	410.0	206.0	160^a^	−110	−10	−38	−13	50	Medium	−4500	500	25	50
D_7_-GAValPFPTH	417.2	234.1	160^a^	−75	−10	−22	−15	50	Medium	−4500	500	25	50

*Q1* quadrupole 1 (*m*/*z*), *Q3* quadrupole 3 (*m*/*z*), *DP* declustering potential (V), *EP* entrance potential (V), *CE* collision energy (V), *CXP* cell exit potential (V), *CUR* curtain gas (psi), *CAD* collisionally activated dissociation gas, *IS* ionspray voltage (V), *TEM* temperature (°C), *GS1* nebulizer gas (psi), *GS2* turbo heater gas (psi)

^a^Dwell time (msec)

^b^Expected retention time (min)

#### Urine analysis [mercapturic acids, urinary creatinine (ucr)]

Assuming a density of 1 kg/L, total urine weight was converted into total urine volume. Urinary creatinine (ucr) was determined in duplicate using an ucr assay kit purchased from Cayman Chemical Company (Ann Arbor, USA) following the manufacturer's instructions. Urinary mercapturic acids were expressed as amounts per excreted total urine volume and normalized to urinary creatinine. Determination of mercapturic acids followed the method published by Ruenz et al. (2016) with some modifications. Urine aliquots (1 mL) were diluted with ammonium formate buffer (50 mM; pH 2.5; 3 mL). As internal standards, D_3_-AAMA (100 ng) and D_3_-GAMA (25 ng) were added. Samples were shaken, pH adjusted to 2.5 with 4 N HCl, and applied to solid-phase extraction (SPE) columns (Isolute ENV+, 100 mg; 10 mL from Biotage, Uppsala, Sweden), preconditioned with methanol (4 mL), bidistilled H_2_O (4 mL), and HCl (pH 2.5; 4 mL). After sample loading, columns were washed with HCl (pH 2.5; 2 mL) and H_2_O/methanol (90/10, v/v; pH 2.5; 1 mL) and subsequently dried under vacuum. Analytes were eluted with methanol/formic acid (99/1, v/v; 1.85 mL) and concentrated to a volume of 100 µL in a vacuum centrifuge. The concentrate was taken up in 0.1% aqueous acetic acid to a volume of 2 mL. After centrifugation (6000 rpm, 10 min), 600 µL 0.1% aqueous acetic acid was added (samples of group A), whereas samples of group B were not diluted further. Samples of 0.1% aqueous acetic acid were analyzed between samples to exclude carry-over effects. Aliquots of 2 µL were injected into a ultra-high performance liquid chromatography (UHPLC)–ESI–MS/MS system (Agilent 1290 Infinity UHPLC/Qtrap 5500 MS) and analyzed using the scheduled multiple reaction monitoring (MRM) mode (MS parameters see Table 1) For HPLC separation, a reversed-phase HPLC column (Agilent Zorbax Eclipse XDB-C18, 50 × 4.6 mm, 1.8 µm) was used, equipped with the corresponding UHPLC guard column. Solvent A: 0.1% aqueous acetic acid; solvent B: acetonitrile; 1% for the first 2 min and then increased up to 10% in 3 min, followed by a reconditioning step. AAMA and GAMA were baseline separated at a flow of 600 µL/min. The respective limit of detection (LOD) and limit of quantification (LOQ) were 0.43 fmol and 0.85 fmol for AAMA and 0.40 fmol and 0.80 fmol for GAMA (absolute amounts). ^13^C_3_D_3_-AAMA was quantified as AAMA, occurrence values below the LOQ were set at 0.

#### Blood analysis (hemoglobin adducts)

Hb adducts were monitored as *N*-terminal valine *N*-(2-carbamoylethyl)-valine (AAVal derived from AA) and *N*-(2-carbamoyl-2-hydroxyethyl)-valine (GAVal derived from GA) by a modified Edman degradation generating pentafluorophenyl thiohydantoin derivatives (AAValPFPTH and GAValPFPTH) which were quantified by UHPLC–ESI–MS/MS. Hb was isolated immediately after blood sampling (BS) as described elsewhere (Schettgen et al. 2003), with slight modifications. About 25 mg of the isolated and dried Hb was weighed and dissolved in 1.5 mL formamide overnight. After derivatization with 20 µL 1 M NaOH and 20 µL pentafluorophenylisothiocyanate (PFPITC), samples were shaken (2 h, 45 °C) and 2 ng of the respective internal standards was added. Proteins were precipitated with 1.6 mL saturated saline solution and diluted with 6 mL H_2_O followed by centrifugation (3000 *g*, 30 min, RT). Supernatants were applied to a SPE column (Isolute ENV+, 100 mg; 10 mL from Biotage, Uppsala, Sweden) preconditioned with 4 mL methanol, 4 mL H_2_O, and 2 mL formamide/water (50/50, v/v). After two washing steps (2 mL H_2_O, 1 mL methanol/water (50/50, v/v) analytes were eluted with 2 mL methanol, concentrated to approximately 150 µL in a vacuum centrifuge, and aliquots of 5 µL were injected into the UHPLC-ESI–MS/MS system (MS parameters see Table 1). HPLC separation was achieved using a Kinetex PFP column (100 × 2.1 mm, 100 A, 2.6 µm, Phenomenex, Aschaffenburg, Germany) equipped with a guard column with 0.1% aqueous acetic acid as solvent A and acetonitrile as solvent B at 40 °C. Acetonitrile concentration was 10%, for 0.5 min, then increased to 40% for 0.6 min, and to 45% for 4.8 min, followed by a reconditioning step.

### Data analysis

MS data were processed by Analyst 1.6 and Multiquant 2.0 Software (AB Sciex). Data analysis and plots were set up with Origin^®^ 2016 G Software (Origin Lab Corporation, Northampton, USA) and SAS V9.1. Data analysis was based on intra-individual differences between each intervention time point to baseline. Due to the small number of volunteers the non-parametric Wilcoxon rank-sum test was used to compare median values between groups and the Wilcoxon signed rank test for differences in the same group. The statistical analysis tested the hypothesis “There are no differences in the median values of the groups” against the two-sided alternative hypothesis “The differences in the median values of the groups are different from zero”. All statistical hypotheses were tested with a 5% significance level. The area under the curve (AUC) was calculated (with c_i_ at the time point t_i_) as follows:

$${\text{AUC}} = \frac{1}{2} \mathop \sum \limits_{i = 1}^{n - 1} \left( {\left( {t_{i + 1} - t_{i} } \right)\; \times \; \left( {c_{i} + c_{i + 1} } \right)} \right)$$

## Results

### Food analysis (acrylamide, caloric intake)

Volunteers of each group consumed the same meal servings during the 13-day human intervention study. Dosimetry of the AA intake on the washout days was achieved as described before and expressed as upper bound AA intake (Ruenz et al. 2016). This was calculated for both groups during washout as 4.2 µg/d equal to 0.05–0.06 µg/kg bw/d. On day 6, group A ingested four cups of coffee, providing 0.15–0.17 µg/kg bw, and on day 10 this group ingested meals providing 14.1–15.9 μg/kg bw of AA (high AA, see Fig. 1; Table 2). On the same day (6), group B ingested isotopically labelled ^13^C_3_D_3_-AA in water (1 μg/kg bw). The estimated daily caloric intake during washout was 2408 ± 83 kcal. The caloric intake on day 10 (high AA, group A) was 2897 kcal (+20%).

**Table 2:** AA contents of food samples with quantifiable AA amounts (mean ± SD; *n* = 3)

Food	AA content (µg/kg)
French fries (lunch)	2778 ± 133
French fries (dinner)	2127 ± 161
Potato crisps	1100 ± 50
Crisp bread	396 ± 23
Coffee pads	241 ± 11
Cereals	182 ± 8
Swedish oat cookies	143 ± 7
Brewed coffee	15.9 ± 0.5

### Urine analysis (mercapturic acids)

**Fig. 2:**
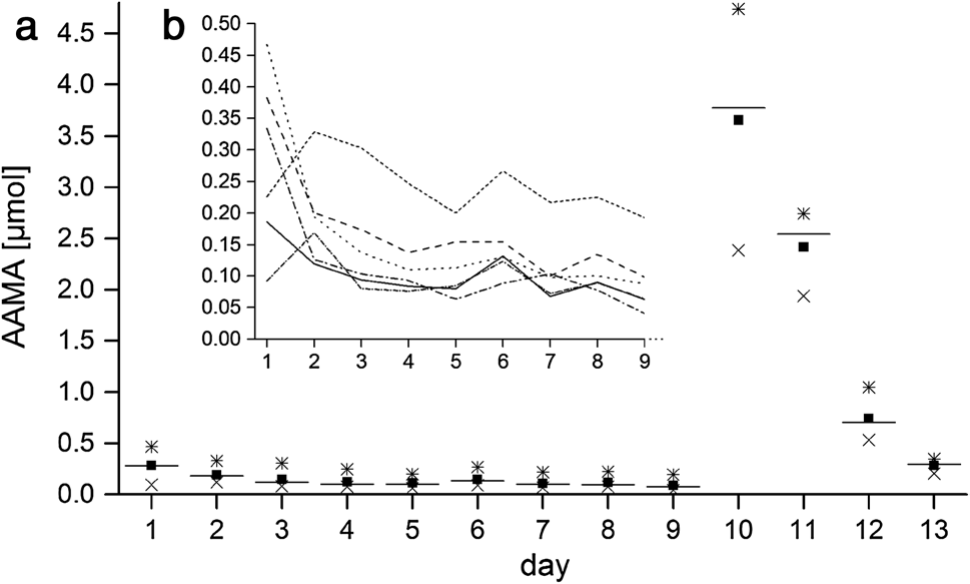
*a* Urinary excretions of AAMA of group A (*n* = 6) for each individual study day (24 h, start at 8 a.m.); squares represent mean values, *horizontal lines* are median values, crosses and stars represent the minimum respective maximum;* b* development of AAMA excretion from day 1 to 9 at the level of each single volunteer of group A indicated as *one line* per volunteer; days 1–5, 7–9, 11–13: washout days (0.05–0.06 µg/kg bw/d), day 6 (coffee): 0.15–0.17 μg/kg bw; day 10 (high AA): 14.1–15.9 μg/kg bw

**Fig. 3:**
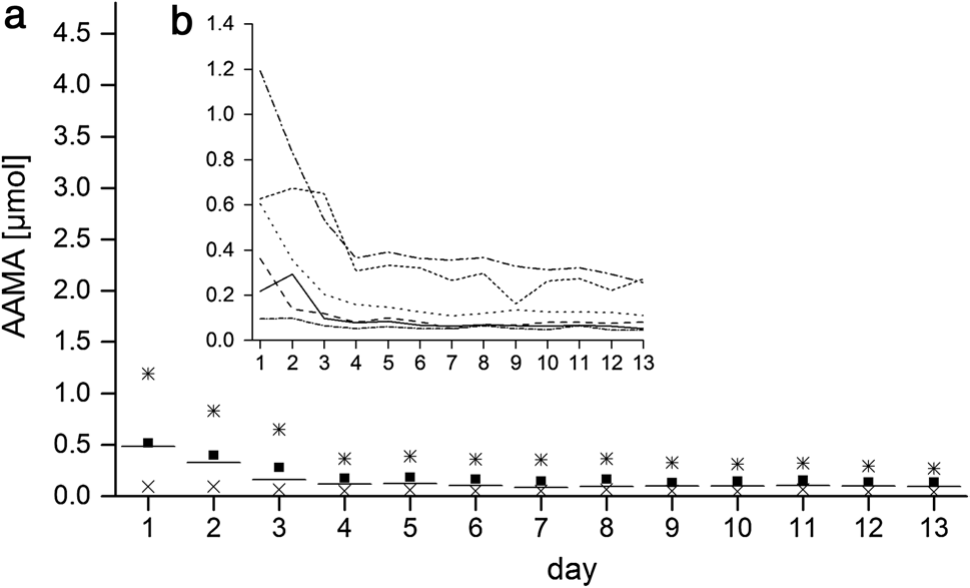
*a* Urinary excretions of AAMA of group B (*n* = 6) for each individual study day (24 h, start at 8 a.m.); squares represent mean values, *horizontal lines* are median values, crosses and stars represent the minimum respective maximum;* b* development of AAMA excretion at the level of each single volunteer of group B indicated as *one line* per volunteer; days 1–13: washout days (0.05–0.06 µg/kg bw/d)

**Fig. 4:**
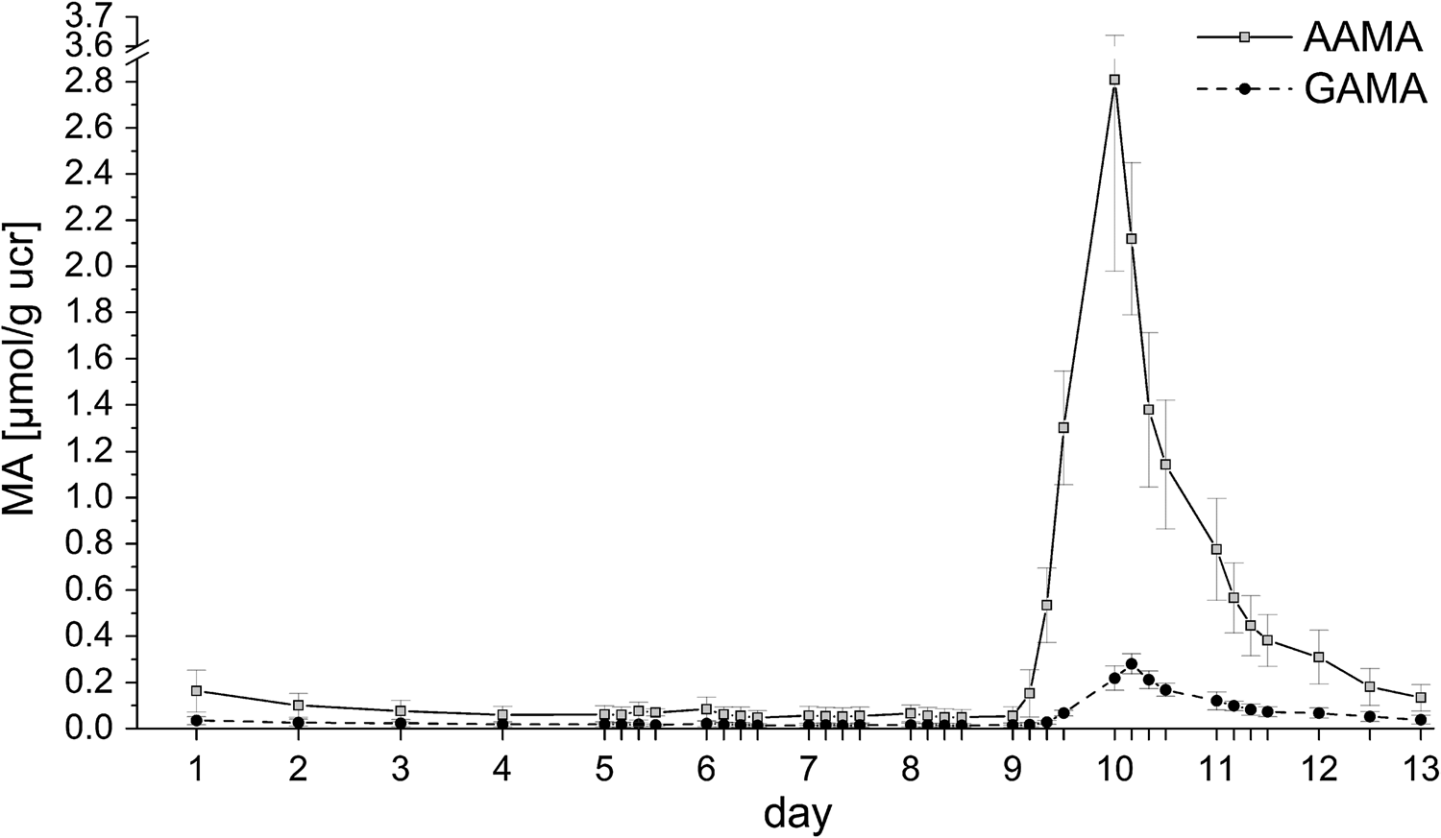
Urinary excretions of AAMA and GAMA (in μmol/g urinary creatinine) of group A for each individual study day (24 h, start at 8 a.m.). Days 1–5, 7–9, 11–13: washout days (0.05–0.06 µg/kg bw/d), day 6 (coffee): 0.15–0.17 μg/kg bw/d; day 10 (high AA): 14.1–15.9 μg/kg bw/d (details see materials and methods section). Data were expressed as mean ± standard deviation (*n* = 6)

During the first washout phase (days 1–5) the AAMA excretion of group A decreased from 0.28 ± 0.14 µmol/d (0.16 ± 0.09 µmol/g ucr) to 0.12 ± 0.05 µmol/d (0.06 ± 0.04 µmol/g ucr) (Fig. 2a). As shown in Fig. 2b the initial individual MA excretion of the volunteers on day 1 ranged from 0.09 to 0.47 µmol AAMA, illustrating different dietary habits prior study start. Until day 9 the mean values of group A were decreasing to 0.09 ± 0.05 µmol/d (range 0.04–0.19 µmol/d). Volunteers of group B were on a higher baseline level at the beginning of the intervention (Fig. 3a) compared to group A, excreting urinary AAMA amounts of 0.52 ± 0.39 µmol (0.29 ± 0.23 µmol/g ucr) on day 1 ranging from 0.10 to 1.19 µmol (see Fig. 3b, note differences in y-scale), continuously decreasing to 0.19 ± 0.14 µmol (0.08 ± 0.06 µmol/g ucr) on day 5. The GAMA excretion (shown in Fig. 4 in relation to creatinine excretion) decreased in group A from 0.06 ± 0.01 µmol (0.03 ± 0.02 µmol/g ucr) on day 1 to 0.03 ± 0.01 µmol (0.02 ± 0.01 µmol/g ucr) on day 5. Values for GAMA in group B were 0.08 ± 0.06 µmol (0.04 ± 0.03 µmol/g ucr) on day 1 and 0.05 ± 0.03 µmol (0.02 ± 0.01 µmol/g ucr) on day 5 (data not shown).

In group A, intake of four portions of 125 mL coffee during 6 h (total AA dose 7.9 µg = 0.15–0.17 µg/kg bw), led to slightly enhanced urinary mean AAMA excretion (0.15 ± 0.06 µmol/d), as compared to 0.12 ± 0.05 µmol/d on the foregoing day 5. Of note, the respective day 5 value (washout) of group B was 0.19 ± 0.14 µmol/d, decreasing towards the end of the study to 0.14 ± 0.10 µmol/d. As seen from Fig. 4, after high AA intake at day 10 (14.1–15.9 µg/kg bw/d), a steep increase of AAMA and GAMA excretion was observed. After 24 h about 23% (15–29%) of ingested AA were excreted, and after 96 h about 44% (38–51%) (Table 3). Corresponding values for GAMA were about 2% (24 h) and about 5% (96 h). Both AAMA and GAMA excretions were still above baseline 96 h after high AA intake via food (Fig. 4) on day 13. AAMA excretion after high dietary intake reached c_max_ (2.79 µmol/g ucr) at 25 h, resulting in an AUC of 6.78 ± 0.74 h × µmol (74.6 ± 14.4 h × µmol/g ucr). The values for GAMA were *t*_max_ = 28 h (*c*_max_ = 0.28 µmol/g ucr) with an AUC of 0.76 ± 0.11 h × µmol (8.6 ± 1.4 h × µmol/g ucr).

**Table 3:** MA excretion given as absolute excreted amounts (in nmol) during the human intervention study with high (14.1–15.9 µg/kg bw) AA intake via food (group A) respective single oral administration of ^13^C_3_D_3_-AA (1 µg/kg bw) of group B

High AA	Washout	AA intake	Washout		
	24 h (day 9)	24 h (day 10)	48 h (day 10–11)	72 h (day 10–12)	96 h (day 10–13)
AAMA (nmol)	91 ± 54	3654 ± 824	5950 ± 780	6811 ± 865	7089 ± 875
GAMA (nmol)	25 ± 12	270 ± 58	628 ± 115	774 ± 133	854 ± 149

^13^C_3_D_3_-AA	^13^C_3_D_3_-AA	Washout			
	24 h (day 6)	48 h (day 6–7)	72 h (day 6–8)	96 h (day 6–9)	120 h (day 6–10)
^13^C_3_D_3-_AAMA (nmol)	303 ± 39	377 ± 52	403 ± 58	412 ± 60	414 ± 62

**Fig. 5:**
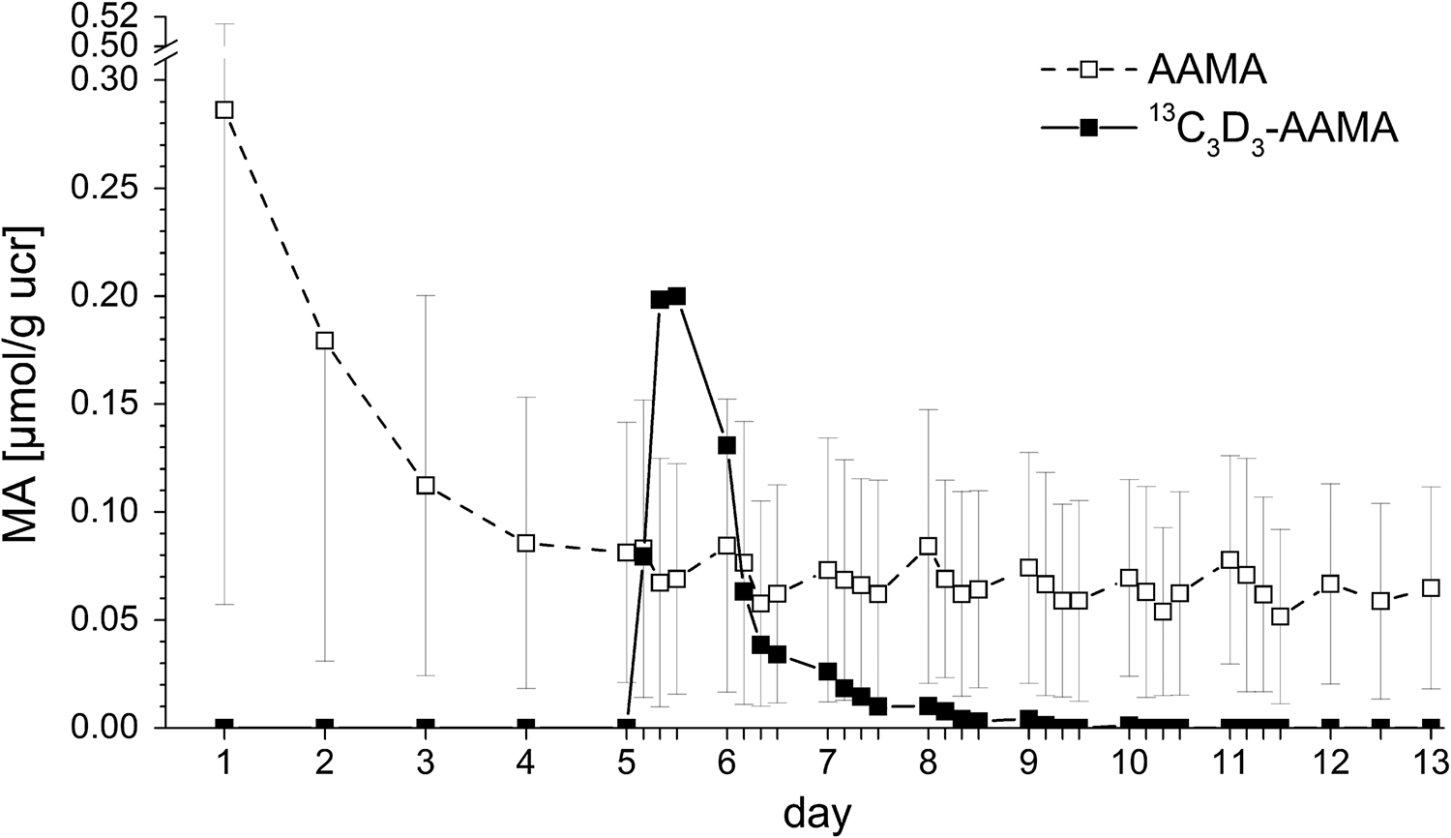
Urinary excretions of AAMA and ^13^C_3_D_3_-AAMA (in μmol/g ucr) for each individual study day (24 h, start at 8 a.m.) of group B. Days 1–13: washout days (0.05–0.06 µg/kg bw/d), day 6 (0 h): single oral administration of 1 μg ^13^C_3_D_3_-AA/kg bw. Data were expressed as mean ± standard deviation (*n* = 6)

As shown in Fig. 5 the isotopically labelled ^13^C_3_D_3_-AA (1 µg/kg bw) was metabolized to the respective labelled mercapturic acid ^13^C_3_D_3_-AAMA. The excretion kinetics of ^13^C_3_D_3_-AAMA indicated that *c*_max_ (0.22 µmol/g ucr) was already reached at *t*_max =_ 11 h. The elimination curve of the isotope labelled AAMA intersected with the unlabelled AAMA background signal about 30 h after intake. Very small traces of ^13^C_3_D_3_-AAMA were still apparent [<LOQ, close to the LOD],  in two out of six urine samples until the end of the study. After 24 h 30% of ^13^C_3_D_3_-AA intake was excreted as ^13^C_3_D_3_-AAMA; after 72 h 40% and after 96 h in total 41% of the ingested ^13^C_3_D_3_-AA were excreted as ^13^C_3_D_3_-AAMA resulting in an AUC of 0.41 ± 0.06 h × µmol (5.1 ± 0.8 h × µmol/g ucr).

### Blood analysis (hemoglobin adducts)

In line with the MA formation, the *N*-terminal valine hemoglobin adduct levels of AA and GA were influenced by dietary AA intake as shown in Table 4. Signals for AAVal as well as GAVal (*p* < 0.05) of group A decreased from initial blood sampling (1. BS, begin of study) to the subsequent sampling time point (2. BS, day 7). Both values responded to high AA intake on day 10 within 3 days (3. BS, day 13; *p* < 0.05). In comparison to group A the volunteers of group B showed higher AAVal and GAVal levels, in accordance with the higher MA excretion at study start (1. BS). In the course of 13 days’ washout, the AAVal and GAVal levels declined at following blood sampling time points (*p* < 0.05).

**Table 4:** *N*-terminal valine hemoglobin adducts of AA (AAVal) and GA (GAVal) during the human intervention study with coffee consumption on day 6 and high AA intake via food on day 10 (group A) respective 13 days of washout (group B)

Blood sampling	AAVal (pmol/g Hb)		GAVal (pmol/g Hb)	
	Group A (*n* = 6)	Group B (*n* = 6)	Group A (*n* = 6)	Group B (*n* = 6)
1. BS	24.5 ± 19.7	46.4 ± 44.1	17.2 ± 16.0	27.5 ± 25.8
2. BS	22.3 ± 17.6	38.4 ± 34.9	15.9 ± 14.5	23.3 ± 21.5
3. BS	26.0 ± 16.1	34.0 ± 32.6	18.7 ± 13.5	20.9 ± 20.7

1. BS prior study start day 1, 2. BS end of day 7, 3. BS end of day 13

## Discussion

The present study was conceived to gain deeper insight into the response of urinary exposure biomarkers with low and high dietary AA intake, ascertained by duplicate diet dosimetry as recommended, e.g. by EFSA (2015). A second aim was to substantiate the evidence for a supposedly consistent background of these biomarkers in humans as indicated by the findings of our foregoing human duplicate diet study (Ruenz et al. 2016) in which a background signal was discovered that was not connected to dietary AA intake. This confirmatory study was to be achieved by exact dosimetry of dietary intake and by using stable isotope tracer methodology. To that end, stable isotope labelled AA was ingested at a dose level close to average dietary exposure (1 µg/kg bw) and urinary biomarker kinetics were monitored. Since no natural background signal for the tracer compound should be expected, clearcut biomarker kinetics at consumer-relevant exposure level of the tracer compound metabolites should be obtained. Monitoring of the labelled biomarker kinetics was also expected to provide a clue to the question whether partial distribution into some deep compartment may cause delayed biomarker release as potential cause for the apparent biomarker background signal. The group B AAMA background signal towards the end of the 13-day washout phase (AA input max. 46–63 ng/kg bw) reached a value of 0.14 ± 0.10 µmol/d (Fig. 3), whereas the corresponding group A value at this day 13 still was somewhat enhanced, most probably due to urinary AAMA tailing from the foregoing very high AA intake on day 10 (Fig. 2a). However, time points under washout, preceding dietary AA ingestion of group A, are also indicative for sustained AAMA background excretion: day 5 (0.12 ± 0.05 µmol) and day 9 (0.09 ± 0.05 µmol), respectively. Taking the excretion of ^13^C_3_D_3_-AA as ^13^C_3_D_3_-AAMA (30% within 24 h) and the maximal dietary AA intake during washout (4.2 µg/d) into account, a net AA background of 0.3 µg/kg bw (day 5) respective 0.2 µg/kg bw (day 9) was calculated. For group B under the same conditions an AA background of 0.3–0.4 µg/kg bw can be estimated on day 13 (0.14 ± 0.10 µmol AAMA). The total AAMA background signal reported in the foregoing study (Ruenz et al. 2016) on day 3 of the initial washout period was 0.09 ± 0.03 µmol at an AA intake of 21–41 ng/kg bw (2.2 ± 0.005 µg/24 h). This corresponded to a net AA background of 0.3–0.4 µg/kg bw/d, too. These values are quite comparable, indicating that at a minimized AA intake (21–63 ng/kg bw/d = 0.3–0.9 nmol/kg bw/d) about 0.09–0.14 µmol/d AAMA is excreted in urine. This over-proportionate urinary MA excretion is reflecting an apparent steady state of endogenous AA background, unrelated to exogenous exposure.

The use of stable isotope labelled AA allowed us to obtain specific and definite information about the excretion kinetics of labelled AAMA in response to an oral AA challenge. After uptake of the labelled AA, the volunteers of group B excreted isotopically labelled AAMA equivalent to 41% of the ingested dose. The kinetics showed a *t*_max_ value of 11 h and *c*_max_ of 0.22 µmol/g ucr. This *c*_max_ and *t*_max_ are in line with data from Ruenz et al. (2016) showing almost complete excretion of AA via AAMA and GAMA within 72 h. The excretion of labelled AAMA intersects with the baseline of unlabelled AAMA excretion 30 h after intake as shown in Fig. 4, thereafter approaching the LOD. This is considered quite compelling evidence against the concept of sustained AA liberation from some deep body compartment, taking into account that AA is very well soluble in water, rapidly distributed throughout the body water compartment, and also rapidly eliminated. Moreover, the chemistry of interaction with cellular nucleophiles like GSH is presumed primarily to follow the rules of the Michael reaction pathway. Nucleophilic attack on the α,β-unsaturated bond results in formation of a covalent thioether bond considered rather resistant against hydrolysis or enzymatic cleavage. The amide carbonyl of AA may not be expected to play a notable role in reacting with cellular nucleophiles, because its reactivity is quenched by the presence of the amide nitrogen. Moreover, MA metabolites are well soluble in water and rapidly eliminated through the urinary tract. A possible explanation for the sustained background of about 0.06 µmol/g ucr may be that Maillard type chemistry also occurs in the living body by continuous reaction of reducing sugars or other carbonyls with amino groups of amino acids, peptides or proteins. This may also generate putative precursor intermediates entailing formation of traces of AA at human body temperature. Such chemistry also relates to the generation of the so-called advanced glycation end products (AGE) in the human body, known to interfere with the function of structural and other proteins (Vistoli et al. 2013).

In this study after high dietary intake of AA (14.1–15.9 μg/kg bw/d) we found 44% (38–51%) of the ingested AA excreted within 96 h as AAMA and 5% as GAMA. This compares with about 58% of ingested AA excreted as AAMA within 72 h in the study of Ruenz et al. (2016), which appears to be a notable difference. Obviously, urinary AAMA excretion at the end of the study period after high intake of around 15 µg/kg bw was not yet complete at this time point. Nonetheless, the AUC value of AAMA (6.78 h × µmol) was about 16 times the AUC value of ^13^C_3_D_3_-AAMA (0.41 h × µmol) after oral administration of 1 µg/kg bw of group B, thus well reflecting the ratio of differential AA intake levels. This applies as well to GAMA, with an AUC of (0.76 h × µmol) equaling about 11% the AAMA AUC. It also deserves consideration that individual AAMA excretion within 96 h ranged between 38 and 51% of the dietary AA intake. Such inter-individual variations between the volunteers may reflect differences in metabolic status and turnover, potentially related in part to enzyme polymorphisms. Of note, the average human dietary AA exposure in Europe is estimated to be 0.4–1.9 μg/kg bw/d (mean) to 0.6–3.4 μg/kg bw/d (95th percentile) (EFSA 2015). The very high dietary AA intake achieved in the present study may reflect certain home cooking procedures supposed to still be in use in European households, such as frying potato chips to deep brown, as done here.

On the other hand, coffee intake of four cups during day 6 revealed a slight, yet still significant increase in AAMA (*p* < 0.01) and GAMA (*p* < 0.05) excretion in the volunteers of group A in relation to the foregoing day 5 in comparison to the respective AAMA and GAMA excretion of group B. Moreover, the comparison to group B shows that the intake of four cups of coffee within one day (0.15–0.17 µg/kg bw) is in the range of the background AAMA excretion of the volunteers on washout. The previous nutrition type and/or lifestyle of the volunteers before entering the study obviously affects to some extent the initial MA background level at start of the washout phase. In addition, two volunteers of group B (Fig. 3b) showed constantly high backgrounds (0.26 and 0.27 µmol/d, respectively) after 13 days of washout. These persons strongly influenced the mean values of AAMA baseline excretion in this group. Without these volunteers the mean background would have been 0.07 ± 0.03 µmol/d, very close to group A (0.09 ± 0.05 µmol). Of note, Wang et al. (2017) excluded four of 110 participants in a human monitoring study due to high AAMA background levels.

Overall, the effect of coffee consumption (500 mL/d) in this intervention study with strictly controlled dietary AA intake did not deviate from the background range of AAMA excretion when groups A and B are considered together. We assume that this may well reflect the situation under uncontrolled dietary situations in normal life. This is supported by results of an ongoing biomarker monitoring study of ‘free living’ people where inter-individual differences in AAMA baseline were found considerably larger than those found here, apparently strongly depending on the individual lifestyle of volunteers even under tightly controlled conditions (publication in preparation).

Hb adducts, by nature of their stability in red blood cells, follow long-term kinetics governed by the lifetime of red blood cells (about 4 months). They, therefore, were not expected to show short-time responses to dietary AA intake challenges, as conceptionalized in the present study. They were, however, expected to provide useful additional information with respect to lifestyle associated longer term AA exposure of volunteers before entering the study. In our study, Hb adduct levels of AA (AAVal) and GA (GAVal) were in line with the findings for urinary AAMA showing a rather broad inter-individual range in both groups. GAVal levels behaved in parallel to AAVal. In group A, no increase was measureable after coffee consumption, whereas some detectable increase was observed after high AA intake in individuals (Table 4). Group B, on washout for 13 days, showed a slight continuous decrease of Hb adducts. Thus, as demonstrated in this study, Hb adducts show a significant response to a substantially enhanced dietary AA intake, however, not to the small intake increments as brought about by coffee consumption. In accordance, the washout regimen resulted in about 22% reduction of Hb levels within 13 days.

## Conclusion

Using enhanced washout periods, this human intervention study corroborates previous findings concerning sustained urinary background excretion of AA exposure-related biomarkers, AAMA and GAMA. Such indications had been found in foregoing animal and human studies (Watzek et al. 2012a; Ruenz et al. 2016). Further support came from the excretion kinetics of stable isotope labelled AA. The labelled mercapturic acid, ^13^C_3_D_3_-AAMA, was practically completely excreted within 72–96 h. The excretion curve intersected with the baseline of sustained background excretion of unlabelled AAMA already 30 h after intake. This clearly excludes the possibility for an explanation of the background signal by distribution and storage of AA (or any other MA precursor) in some deep body compartment.

Altogether, this study provides compelling support for the hypothesis of a sustained endogenous AA formation in the human body. Moreover, whereas a high dietary AA intake (14.1–15.9 μg/kg bw/d) provided prominent biomarker responses, urinary biomarker response after ingestion of four cups of coffee (total AA dose 7.9 µg = 0.15–0.17 µg/kg bw) became just detectable by individual excretion kinetics. However, this small response did not deviate from the overall inter-individual background range of all volunteers on washout. This result clearly indicates normal coffee consumption at best to contribute to a minor degree to overall dietary AA exposure.
